# Knowledge About Basic Life Support Among University Students

**DOI:** 10.7759/cureus.76293

**Published:** 2024-12-24

**Authors:** Rita Cruz, Pedro Lito

**Affiliations:** 1 Critical Care Unit, Unidade Local de Saúde São José, Lisbon, PRT; 2 Critical Care Unit, Unidade Local de Saúde da Cova da Beira, Covilhã, PRT

**Keywords:** adult cardiac arrest, automatic external defibrillator, basic life support (bls), cardiac arrest outcome, out-of-hospital cardiac arrest

## Abstract

Background: Basic life support (BLS) is an essential skill set for responding to emergencies like cardiac arrest. However, the level of preparedness and interest in BLS among university students remains underexplored, especially in nonmedical populations.

Methods: This study surveyed 427 University of Beira Interior (UBI) students to assess their knowledge, confidence, and interest in BLS training. A cross-sectional questionnaire was used to gather data on previous BLS exposure, perceived competence, and preferences for further training.

Results: This study focused on a sample of 427 students from the UBI, where 246 (57.6%) had BLS training (group A), predominantly from health-related courses, while 181 (42.4%) had no such training (group B), primarily from non-health-related courses. The study revealed that students with BLS training had significantly higher confidence and preparedness in recognizing and responding to emergencies, such as performing cardiopulmonary resuscitation and using defibrillators, compared to those without training. Despite the overall interest in BLS training, particularly through practical sessions, there remains a disparity in access to such education, particularly among students in non-health-related fields. The findings highlight the need for integrating BLS training into the curriculum, potentially starting at the secondary education level, to ensure widespread competence in life- saving skills across the population.

Conclusions: The study highlights the critical need for integrating BLS training into earlier stages of education to improve preparedness across the population. The findings suggest that widespread, practical, and accessible BLS training could significantly enhance emergency response outcomes.

## Introduction

Cardiovascular diseases remain the leading cause of death globally, with sudden cardiac arrest being a significant contributor to mortality rates. Immediate and effective basic life support (BLS) significantly improves survival chances during such emergencies. BLS encompasses vital techniques like cardiopulmonary resuscitation (CPR) and the use of automated external defibrillators (AEDs), which can double or triple the likelihood of survival if administered promptly [1-5].

Despite the critical importance of BLS, public awareness and competence in these life-saving techniques remain inadequate. In the United States, only 39.2% of out-of-hospital (OOH) cardiac arrests receive BLS, and only 11.9% of these involve the use of an AED. The probability of survival in an OOH setting, where the situation is not correctly identified when calling the emergency number, is 5%. Around half of cardiac arrest cases occur in an OOH setting, with 66% of these happening at home. Although the connection between CPR maneuvers and the victim's survival has been proven, these maneuvers are applied in only 30% of CPR cases. Various studies indicate that bystander intervention rates are low, often due to a lack of knowledge, fear of causing harm, or anxiety about legal repercussions. As such, BLS training has traditionally been emphasized in healthcare settings. However, its importance is increasingly recognized in the general population, particularly among young adults in educational institutions [1-3,6-8].

University students, a demographic that includes future professionals in various fields, represent a key target group for BLS education. However, there is limited research on the level of BLS knowledge and the willingness to undergo training within this population, particularly in non-medical universities. The University of Beira Interior (UBI), with its diverse student body, provides a valuable case study to explore these aspects [2]. The list of courses is attached in the Appendix.

This study aims to evaluate the existing knowledge of BLS among UBI students, assess their confidence in performing BLS, and gauge their interest in further training. Furthermore, the study seeks to explore the potential for integrating BLS training into high school education (peer training, video and self-learning, in-person or not), which could serve as a foundational step in building a more prepared and responsive society [9-11].

## Materials and methods

Creation of the survey

The questionnaire was developed through multiple stages and iterations. Initially, it was reviewed by three sixth-year medical students with certified BLS training to assess question relevance, leading to modifications. Two experienced BLS instructors then reviewed the revised version to ensure scientific accuracy and validity. A group of 29 untrained volunteers later provided feedback to identify unclear questions. The final version, created with input from BLS specialists, was divided into sections addressing social background, knowledge perception, individual status, actions to take, and interest in training.

The questionnaire was designed to capture a comprehensive view of students' BLS knowledge, prior training, and their perceived competence in performing BLS techniques. Additionally, the survey included questions about students' interest in receiving further training and their opinions on the integration of BLS education into high school curricula.

The questionnaire, available in the Appendix, was structured into four main sections. The first section gathered demographic data, including age, gender, field of study, and academic level. The second focused on prior exposure to BLS training, asking about the type, recency, and context of the training. The third section involved a self-assessment of perceived competence and confidence in performing BLS techniques such as CPR and AED use. The final section explored interest in further training, including preferences for instructional formats and the importance of making BLS training mandatory in secondary education.

Ethics committee approval

After the development of the questionnaire, the research project was submitted to the Ethics Committee of the UBI and approved under the code CE-UBI-Pj-2018-078.

Study design and participants

This observational, cross-sectional study was conducted at UBI in Covilhã, Portugal, from January to February 2019. The target population included undergraduate and graduate students across various faculties, totaling 7,400 students. The sample includes 427 volunteers who were divided into two main groups: group A, students from UBI who received BLS training 246 (57.6%), and group B, students from UBI who did not receive BLS training 181 (42.4%).

Statistical analysis

The collected data were analyzed using descriptive statistics to summarize the participants' demographic characteristics, previous BLS training, and perceived competence. Chi-square tests were used to examine the association between previous training and perceived confidence in performing BLS. Logistic regression analysis was performed to identify predictors of interest in further BLS training. Statistical significance was set at p < 0.05.

## Results

Demographic characteristics

The study sample consisted of 427 students with a median age of 22 years. The gender distribution was approximately balanced, with 222 (52%) female and 205 (48%) male participants. The majority of respondents were undergraduate students 333 (78%), with the remaining 94 (22%) being graduate students. The participants were drawn from various faculties, including health sciences 128 (30%), engineering 107 (25%), social sciences 85 (20%), and other disciplines 107 (25%) (Table 1).

**Table 1:** Overview of the demographic characteristics (n = 427)

Category	n (%)
Gender distribution	
Female	222 (52)
Male	205 (48)
Age distribution	
≤22 years	284 (66.5)
>22 years	143 (33.5)
Education level	
Undergraduate students	333 (78)
Graduate students	94 (22)
Faculty distribution	
Health sciences	128 (30)
Engineering	107 (25)
Social sciences	85 (20)
Other disciplines	107 (25)

Previous BLS training

Based on previous BLS training and the university course in which they were enrolled, the population was divided into two groups and two subgroups, as illustrated in Table 2. Of the total respondents, 246 (57.6%) reported having received previous BLS training. The majority of these students had received training within the last two years, 159 (64.8%), while the remaining 87 (35.2%) had been trained more than two years ago. Most of the training was conducted in formal educational settings, 111 (45%), followed by extracurricular activities, 74 (30%), and workplace training, 61 (25%), as illustrated in Table 2.

**Table 2:** Previous BLS training (n = 427) BLS: basic life support; UBI: University of Beira Interior

Group	Description	n (%)
A	Students at UBI with BLS training	246 (57.6)
A1	Students at UBI with BLS training enrolled in a health-related course	217 (50.9)
A2	Students at UBI with BLS training enrolled in a non-health-related course	28 (6.7)
B	Students at UBI without BLS training	181 (42.4)
B1	Students at UBI without BLS training enrolled in a health-related course	69 (16.2)
B2	Students at UBI without BLS training enrolled in a non-health-related course	112 (26.2)

Knowledge of BLS training

In the six questions of the questionnaire aimed at assessing knowledge in BLS (from scenario recognition to performing the various maneuvers), the differences between groups A and B were statistically significant, with p ≤ 0.001 in the chi-square test. The same phenomenon was not observed when comparing the responses of subgroups B1 and B2 (Table 3).

**Table 3:** p value and chi-square analysis of the association between groups A and B, and between subgroups B1 and B2 for each question

Question	Groups A and B		Groups B1 and B2	
	p value	Chi-square	p value	Chi-square
7	≤0.001	147.002	0.006	7.606
8	≤0.001	21.574	0.675	0.176
9	≤0.001	67.104	0.413	0.671
10	≤0.001	151.084	0.427	0.630
11	≤0.001	181.892	0.038	4.284
12	≤0.001	194.361	0.8	0.060

Perceived competence and confidence

Among students with prior BLS training, 242 (98.4%) reported feeling capable in their ability to perform BLS. In contrast, only 54 (29.8%) of untrained students felt confident in performing these techniques. The disparity in perceived competence was significant, with trained students consistently rating their ability to perform CPR, use an AED, and manage an unconscious person higher than their untrained peers (Qui-quadrado = 275,422 and p < 0.001), as shown in Table 4.

**Table 4:** Assessment of BLS knowledge among questionnaire participants, comparing the group with BLS training (group A) and the group without BLS training (group B) BLS: basic life support

Likert scale	Group A, n (%)	Group B, n (%)	
		Group B1	Group B2
I have no knowledge	0 (0)	2 (2.9)	25 (22.3)
I have little knowledge	4 (1.6)	28 (40.6)	72 (64.3)
I have some knowledge	78 (31.7)	37 (53.6)	15 (13.4)
I have a lot of knowledge	118 (48.0)	1 (1.4)	0 (0)
I have full knowledge	46 (18.7)	1 (1.4)	0 (0)
Total	246 (100)	69 (100)	112 (100)

Interest in further training

A significant proportion of students, 402 (94.1%), expressed interest in receiving further BLS training. For these 402 students, questions with a Likert scale were used to identify their preferred instructional format. Practical, hands-on workshops emerged as the most favored method, with 257 respondents (60.2%) fully agreeing. This was followed by self-directed learning modules (169; 42%), theoretical classes (113; 28%), and video tutorials (103; 25.6%), as shown in Table 5. Additionally, in a question posed to all 427 participants, 415 respondents (97%) supported making BLS training mandatory in high school.

**Table 5:** Distribution of the preferred training type according to the Likert scale among the respondents

Likert scale	Practical, n (%)	Theoretical, n (%)	Video tutorials, n (%)	Self-directed, n (%)
Would not like at all	0 (0)	6 (1.5)	9 (2.2)	3 (0.8)
Would not like	2 (0.5)	25 (6.2)	36 (8.9)	19 (4.7)
No opinion	5 (1.2)	33 (8.2)	58 (14.4)	33 (8.2)
Would like	138 (32.3)	225 (56)	196 (48.7)	178 (44.3)
Would like very much	257 (60.2)	113 (28)	103 (25.6)	169 (42)
Total	402	402	402	402

## Discussion

This study examined knowledge and interest in BLS training among 427 students at the UBI. The findings revealed that 57.6% of the participants (group A), primarily from health-related courses, had received BLS training, while 42.4% (group B), mostly from non-health-related fields, had no prior training.

Students with BLS training demonstrated significantly higher confidence and preparedness to recognize and respond to emergencies, such as performing CPR and calling emergency services. These results align with existing literature, which highlights the effectiveness of BLS training in enhancing readiness to act in critical situations. For instance, the effectiveness of online and in-person BLS training in improving knowledge and practices was evaluated, concluding that both formats significantly contributed to increased knowledge and self-confidence [12].

Conversely, students without BLS training, including those in health-related fields at earlier stages of their studies, exhibited critical gaps in these competencies. This underscores the need to incorporate BLS training into the curricula of health-related programs from the early years. The level of knowledge and motivation for BLS training in the school community were analyzed, which also identified that 90.1% of participants lacked any BLS training, emphasizing the necessity of systematic educational approaches [13].

The significant interest in receiving BLS training, expressed by 94.1% of respondents, and the willingness of 63.2% to pay for this training demonstrate the recognition of its importance. The preference for practical sessions aligns with evidence, suggesting that hands-on learning is the most effective approach for consolidating skills and improving knowledge retention [12].

The strong support for integrating BLS training into high school curricula observed in this study is consistent with the literature, as highlighted in the review by Bray et al., which emphasizes the need for early and structured resuscitation education to improve community response and survival rates in emergencies [14].

Additionally, literature from other contexts highlights that ongoing education in BLS is essential for maintaining skills and knowledge over time. The impact of permanent training programs in BLS and advanced cardiovascular life support on the knowledge of healthcare professionals was evaluated, which reported a significant increase in knowledge levels, with gains of up to 91% in the total sample [15].

The disparities observed between students with and without prior BLS training in this study underscore the need for a more inclusive and comprehensive approach to education, ensuring that all individuals, regardless of their background or training status, have access to essential life-saving skills. Integrating BLS training broadly across disciplines and educational levels could bridge these gaps, promoting equity in access to essential life-saving skills. This study contributes to the ongoing discourse on the critical need for the widespread implementation of BLS education to improve public health and safety.

Limitations of the study

While this study provides valuable insights, it is important to acknowledge its limitations. The cross-sectional design captures a snapshot of student knowledge and interest at a single point in time, which may not fully reflect long-term trends or changes in attitudes. Additionally, the study relies on self-reported data, which may be subject to biases such as overestimation of competence or interest in training. Future research should consider longitudinal studies to track changes in BLS knowledge and training over time and explore the impact of different educational interventions.

## Conclusions

This study highlights the critical need for enhanced BLS training among university students, particularly those without prior exposure to life-saving techniques. The findings underscore the importance of practical, hands-on training and support the integration of BLS education into secondary school curricula. By equipping young adults with the knowledge and confidence to act in emergencies, we can significantly improve public health outcomes and save lives. Further research is needed to explore the most effective strategies for implementing BLS training in educational settings and to assess the long-term impact of these interventions on emergency response behavior.

## Appendices

**Table 6:** List of University of Beira Interior courses

List of University of Beira Interior courses
PhD in Philosophy
PhD in Physics
PhD in Management
PhD in Marketing and Strategy
PhD in Mathematics and Applications
PhD in Advanced Materials and Processing
PhD in Media Arts
PhD in Medicine
PhD in Psychology
PhD in Chemistry
PhD in Sociology
Postgraduate in Communication and Science Management
Postgraduate in Humanitarian Crises and International Cooperation
Postgraduate in Physics for Teachers
Postgraduate in Project Management and Family Business Finance
Postgraduate in Hydrology and Climatology
Postgraduate in Master in Business Administration
Postgraduate in Tele-Health
Postgraduate in Regenerative Therapies
Master's in Pedagogical Supervision
PhD in Biomedicine
PhD in Biochemistry
PhD in Science and Engineering of Fibrous Materials
PhD in Political Science
PhD in Communication Sciences
PhD in Sports Science
PhD in Pharmaceutical Sciences
PhD in Fashion Design
PhD in Economics
PhD in Aeronautical Engineering
PhD in Civil Engineering
PhD in Paper Engineering
PhD in Industrial Engineering and Management
PhD in Electrical and Computer Engineering
PhD in Computer Engineering
PhD in Mechanical Engineering
PhD in Textile Engineering
PhD in Communication Studies: Technology, Culture, and Society
Bachelor's in Bioengineering
Bachelor's in Biochemistry
Bachelor's in Biotechnology
Bachelor's in Political Science and International Relations
Bachelor's in Biomedical Sciences
Bachelor's in Communication Sciences
Bachelor's in Sports Science
Bachelor's in Cinema
Bachelor's in Cinema
Bachelor's in Fashion Design
Bachelor's in Industrial Design
Bachelor's in Multimedia Design
Bachelor's in Economics
Bachelor's in Industrial Engineering and Management
Bachelor's in Electromechanical Engineering
Bachelor's in Electrical and Computer Engineering
Bachelor's in Computer Engineering
Bachelor's in Portuguese and Spanish Studies
Bachelor's in Management
Bachelor's in Web Computing
Bachelor's in Marketing
Bachelor's in Optometry and Vision Sciences
Bachelor's in Psychology
Bachelor's in Industrial Chemistry
Bachelor's in Medicinal Chemistry
Bachelor's in Sociology
Bachelor's in Information Technology and Systems

**Table 7:** Questionnaire UBI: University of Beira Interior; BLS: basic life support; CPR: cardiopulmonary resuscitation; AED: automated external defibrillator

Section	Question	Response options
Social background	1. In which of the following age groups do you fall?	≤22 years
		>22 years
	2. Select your gender	Female
		Male
		Prefer not to say
	3. Select the course you are enrolled in at the University of Beira Interior	List of UBI courses at that date
	4. Is this your first Higher Education course?	Yes
		No
	5. Have you ever participated in a BLS course?	Yes
		No
Perception of knowledge level	6. How do you rate your knowledge of BLS?	I have no knowledge of BLS
		I have little knowledge of BLS
		I have some knowledge of BLS
		I have much knowledge of BLS
		I have full knowledge of BLS
Perception of the individual’s condition	7. What is the first step when you find an unresponsive person?	Check for pulse
		Call for help
		Check for responsiveness
		Check if the area is safe
		I don’t know
	8. In which of the following situations do you consider it essential to start CPR?	Man, 30 years old, fainted while watching a football match, exposed to the sun during peak heat, pale, responding slowly
		Woman, 45 years old, found unconscious
		Woman, 22 years old, fainted while running, not communicating, no signs of breathing
		Man, 50 years old, complaining of chest and left arm pain, breathing rapidly
		I don’t know
Actions to be taken	9. During a call to the Medical Emergency Service (112), which of the following actions is not correct?	Provide the location of the accident
		Describe what happened and how it happened
		Give information about the victim's identity and condition
		Hang up as soon as possible to free the line
		I don’t know
	10. After confirming the need for BLS in an adult, which of the following maneuvers should we start with?	Compressions
		Ventilations
		Place the individual in the safety side position
		Place the victim in a comfortable position
		I don’t know
	11. A cycle of compression and ventilation in an adult consists of	15 compressions + 1 ventilation
		15 compressions + 2 ventilations
		30 compressions + 1 ventilation
		30 compressions + 2 ventilations
		I don’t know
	12. About the use of an AED	It should be used as soon as the device is available
		It can only be used after two full cycles of compression and ventilation
		No maneuver can be started until the AED arrives
		The AED can only be used if there are two people providing assistance
		I don’t know
Interest in training	13. Would you be interested in receiving training in this area for free? (If the answer to 13 is No, skip to question 20)	Yes
		No
	14. Would you be interested in receiving training in this area for a fee?	Yes
		No
	15. How much would you be willing to pay for access to the training?	Up to €20
		Up to €50
		Up to €100
		More than €100
	16. Would you like to have practical classes in this area?	Not at all
		Wouldn't like
		No opinion
		Would like
		Would like very much
	17. Would you like to have face-to-face theoretical classes in this area?	Not at all
		Wouldn't like
		No opinion
		Would like
		Would like very much
	18. Would you like to have video-based classes in this area?	Not at all
		Wouldn't like
		No opinion
		Would like
		Would like very much
	19. Would you like to have access to self-learning materials in this area?	Not at all
		Wouldn't like
		No opinion
		Would like
		Would like very much
	20. Would you have liked to learn about these topics in Secondary Education?	Not at all
		Wouldn't like
		No opinion
		Would like
		Would like very much
	21. Do you think it would have been important to have learned about these topics in Secondary Education?	Not important at all
		Not important
		No opinion
		Important
		Very important
